# Calcium and postoperative atrial fibrillation: round up the usual suspects!

**DOI:** 10.1093/cvr/cvab185

**Published:** 2021-05-29

**Authors:** Barbara Casadei, Karin R Sipido

**Affiliations:** 1 Division of Cardiovascular Medicine, BHF Centre of Research Excellence, John Radcliffe Hospital, University of Oxford, Oxford OX3 9DU, UK; 2 Department of Cardiovascular Sciences, Experimental Cardiology, KU Leuven, Leuven, Belgium

**This editorial refers to ‘Altered atrial cytosolic calcium handling contributes to the development of postoperative atrial fibrillation’ by FE. Fakuade *et al.*, pp. 1790–1801**

Postoperative atrial fibrillation (AF) is a frequent and burdensome complication in patients undergoing coronary revascularization and heart valve surgery. Whilst it is recognized that haemodynamic stress and the inflammatory response associated with cardiac surgery may play an important role in triggering the arrhythmia, patients with a diagnosis of postoperative AF who are discharged from hospital in sinus rhythm are more likely to develop AF and ischaemic stroke on long-term follow-up.^1^^,^^2^ Such findings imply that paroxysmal AF episodes brought about by acute illness or stress may still reflect the presence of a pre-existing substrate^3^^,^^4^ subtending future arrhythmia, myocardial dysfunction and cardioembolic complications.

To date, atrial electrical remodelling and fibrosis have been a main focus of research in AF, even though both factors are more robustly identifiable as a consequence rather than a primary cause of AF. In contrast, large prospective cohorts have identified impaired left atrial function (i.e. a lower emptying fraction) in individuals in sinus rhythm as an independent predictor of incident AF and ischaemic stroke.^5^^,^^6^ As for all observational findings, whether or not left atrial function is causally associated with AF (vs. being a biomarker of other known or unknown AF or stroke risk factors)] remains to be conclusively established, as do the mechanisms by which reduced atrial contraction may lead to AF.

In this issue of *Cardiovascular Research*, Fakuade *et al*.^7^ report a reduction in calcium transient amplitude and intracellular stores, and slower rate of calcium reuptake in atrial myocytes isolated from the right atrial appendage of patients in sinus rhythm who develop AF after cardiac surgery compared with those who did not. These findings would be expected to result in impaired contraction and indeed in a subgroup of patients, the authors show that preoperative left atrial systolic performance in patients who develop AF after surgery is lower than that recorded in those who maintain sinus rhythm postoperatively.

Based on our current knowledge of the mechanisms regulating intracellular calcium handling in cardiomyocytes, one could easily identify a number of ‘usual suspects’ that may account for this phenotype, some of which were swiftly acquitted. For instance, differences in postoperative heart rhythm were not associated with changes in peak and overall calcium influx through the L-type calcium channel (under basal conditions or in the presence of isoprenaline) or with differences in intracellular calcium buffering properties, t-tubule density, sodium-calcium exchanger (NCX) function or sarcolemmal calcium ATP-ase activity. Importantly, differences in ryanodine receptor (RyR) phosphorylation or calcium leak did not account for these findings.

A slower rate of intracellular calcium decay in paced cardiomyocytes in the absence of differences in NCX function would point to a reduced SR calcium-ATPase (SERCA2A) activity. SERCA2A is the master regulator of intracellular calcium cycling and its activity is chiefly responsible for the rate of calcium reuptake in the SR, which in turn influences the calcium buffering reserve of contractile proteins and the intracellular calcium stores, and plays an important role in both cardiomyocyte contraction and relaxation, particularly under unloaded conditions.

The relationship between SERCA2A activity and arrhythmia is more complex and may vary depending on concomitant changes in diastolic calcium and the threshold for SR calcium leak/release from the RyR. In previous publications, SERCA2A activity has been reported to be higher in atrial myocytes from patients with postoperative or paroxysmal AF^8^^,^^9^ and the combination of raised RyR phosphorylation and increased SERCA2A activity has been recently shown to increase the probability of AF induction in Langendorff-perfused, aged mouse hearts.^10^ Even in the presence of severe heart failure, SERCA2A expression has not been shown to be reduced in human atrial myocytes^11^ and, taking all evidence into consideration, one would conclude that a difference in SERCA2A activity, when present, is more commonly ascribed to its modulation by regulatory peptides or by post-translational modifications than explained by differences in its total protein content. Here, however, the atrial phenotypic differences in patients who develop postoperative AF were attributed to a reduction in SERCA2A protein. Whilst the average data show a significantly lower SERCA2A protein content in right atrial appendage homogenates of patients who develop postoperative AF, the reduction is modest and the overlap between groups considerable. Phospholamban, the most comprehensively studied of all SERCA2A inhibitory peptides, and its serine 16 and threonine 17 phosphorylated fractions were not reported as such but only as ratios to SERCA2A with no difference between groups.

Could these changes in SERCA2A protein content, in the absence of proportional differences in total and phosphorylated phospholamban, be sufficient to explain the reported abnormalities in intracellular calcium handling? Whilst the findings cannot conclusively prove or disprove that is the case, the authors duly noted that, in addition to phospholamban, SERCA2a activity is modulated by a host of other proteins,^12^ amongst which the SERCA2A inhibitors sarcolipin and calreticulin are known to be expressed in the human atrial myocardium and be reduced in the presence of AF. Sarcolipin mRNA levels did not differ significantly between groups (nor did SERCA2A mRNA levels, Online Figure VI), but whether the protein is increased or decreased under these conditions remains to be seen. Similarly, investigation of the post-translational modifications of SERCA2A (e.g. phosphorylation, S-glutathionylation, SUMOylation and others^12^) might shed further light on the fate of the protein and the regulation of its activity.

In the absence of an increase in diastolic calcium and RyR calcium leak, how might a reduction in SERCA2A activity promote postoperative AF? The authors suggest this may occur through the promotion of calcium transient and action potential alternans, as predicted *in silico* and demonstrated experimentally. Alternans in cardiac myocytes typically manifests as fluctuations in action potential duration, calcium transient amplitude, and contraction amplitude. Both *in silico* models and clinical investigations have shown that cardiac alternans may play an important role in the initiation and maintenance of AF^13^ but, again, to which extent calcium-induced atrial alternans may be driven by changes SERCA2A activity rather than, as previously described, by the properties of RyR remains unclear. Other factors currently not taken into account are the cell-cell-coupling and tissue distribution, which will require more complex models.

Whilst a lot has been learnt from studying atrial myocytes isolated from samples of the right atrial appendage taken before cardiopulmonary bypass, postoperative AF occurs after reperfusion when local and systemic oxidative stress^14^ and inflammatory response to surgery are at their peak. How these phenomena interact with the pre-existing atrial substrate to conjure up the arrhythmia (and by which mechanism) is still a matter of speculation.

Humans are the ultimate ‘model’ of postoperative AF and the authors are to be commended for collecting data from a large number of patients. Yet, even the ultimate model is prone to limitations that are also visible in this study, some of which are unavoidable; e.g. small tissue samples precluding the attainment of matched data for related mechanisms, as illustrated in Online Figure 1. Others, such as the large variation between patients and in the success of cell isolation could have been mitigated by the use of more advanced statistics.^15^

In summary, in a series of carefully conducted experiments in human atrial myocytes Fakuade *et al*.^7^ have confirmed that a subtle atrial cardiomyopathic phenotype comprising of abnormal calcium handling, reduced contraction, and increase alternans precedes the new onset of AF after cardiac surgery. Whilst, as it is always the case, many questions remain unanswered, this evidence adds another layer to the complexity of the mechanisms underpinning the new onset of AF in patients and uncovers potential new targets for its prevention and treatment.

## Funding

BC is funded by the British Heart Foundation (CH/12/3/29609).

**Conflict of interest:** none declared.

## Notes

The opinions expressed in this article are not necessarily those of the Editors of *Cardiovascular Research* or of the European Society of Cardiology.
